# Supplementary material for: Morphinan Alkaloids and Their Transformations: A Historical Perspective of a Century of Opioid Research in Hungary
Source: Int J Mol Sci. 2025 Mar 18;26(6):2736. doi: 10.3390/ijms26062736 (PMC11943231; doi:10.3390/ijms26062736)
Supplement: Supplementary file 1 [file ijms-26-02736-s001.zip › ijms-3500608-supplementary.pdf]

Suplementary Material

Morphinan Alkaloids and Their Transformations: A Historical Perspective of a Century of Opioid Research in Hungary [§]

CONTENTS

		Page
**1.**	**Table S1** Abbreviations	3–4
**2.**	**Figure S1**. Morphine alkaloid research group of the Kossuth Lajos Univesity (KLTE) in Debrecen and Alkaloida Chemical Company, Tiszavasvári, Hungary	5
**3.**	Scientific titles awarded by the University of Debrecen in the field of poppy alkaloids	6–10

1. Table S1 ABBREVIATIONS

Compound, Term, Acronym	Comp.	Name, Synonyms
**AC**	**-**	adenyl cyclase
**ACh**	**-**	acetylcholine; CAS-RN: [51-84-3]
**3-*O*-Ac-$[^{18}F]$cyF**	**$[^{18}F]$203a**	3-*O*-$[^{18}F]$acetylcyclofoxy; 3-*O*-Acetyl-6-deoxy-6-beta-$[^{18}F]$fluoronaltrexone
**ADDP**	**-**	1,1′-(azodicarbonyl)dipiperidine; CAS-RN: [10465-81-3]
**Azidomorphine**	**130**	6-AM; 6-desoxy-6-azidodihydroisomorphine; 3-hydroxy-4,5-*α*-epoxy-6β-azido-7,8-dihydro-17-methylmorphinan; CAS-RN: [22952-87-0]
**Azidoethylmorphine**	**144**	"3-*O*-ethylazidomorphine", azidodionine; 6-azido-6-desoxy-ethyldihydroisomorphine; CAS-RN: [54301-21-2]
**Azidopholcodine**	**145**	3-*O*-morpholinylethyl-azidomorphine; (5*α*,6β)-6-azido-4,5-epoxy-17-methyl-3-[2-(4-morpholinyl)ethoxy]-morphinan; CAS-RN: [54350-46-8]
**BBB**	**-**	blood brain barrier
**BIA**	**-**	benzylisoquinoline alkaloid
**BPN**	-	buprenorphine, CAS RN: [52485-79-7]
**BrCN**	**-**	cyanogen bromide, CAS RN: [506-68-3]
**CAM**	**149a**	*N*^{17}-cyclopropylmethyl-norazidomorphine; (5*α*,6β)-6-azido-4,5-epoxy-17-cyclopropylmethyl-morphinan-3-ol; CAS-RN: [56337-94-1]
**CNM**		isolated nictitating membrane of the cat
**COAM**	**-**	*N*^{17}-cyclopropylmethyl-14-hydroxynorazidomorphine; (5*α*,6β)-6-azido-17-cyclopropylmethyl-4,5-epoxy-morphinan-3-14-diol; CAS-RN: [61266-87-3]
**CNS**	**-**	central nervous system
**CPM**	**-**	cyclopropylmethyl group
**cyF**	**204c**	cyclofoxy; 6-deoxy-6β-fluoro-noroxymorphone; 17-cyclopropylmethyl-4,5*α*-epoxy-6β-fluoro-morphinan-3,14-diol; CAS-RN: [103233-57-0]
**$[^{11}C]$cyF**		$[^{11}C]$cyclofoxy
**CYP450**		cytochrome P450
**Cypre**	**-**	cyprenorphine, RX285M, 17-cyclopropylmethyl-4,5-epoxy-3-hydroxy-6-methoxy-*α*,*α*-dimethyl-6,14-ethenomorphinan-7-methanol,<br>CAS RN: [44065-22-8]
**DA**	**-**	dopamine; CAS-RN: [51-61-6]
**DAST**	**-**	diethylaminosulfur trifluoride; CAS-RN: [38078-09-0]
**DA reaction**	**-**	Diels-Alder reaction
**DEAD**	**244**	diethyl azodicarboxylate, CAS RN: [1972-28-7]
**dihydrothevinone**	**181**	4,5*α*-epoxy-18,19-dihydro-17-methyl-3,6-dimethoxy-7*α*-acetyl-6,14-ethenomorphinan, CAS RN: [16196-82-0]
**DIAD**	**244**	diisopropyl azodicarboxylate; CAS-RN: [2446-83-5]
**DMF**	**-**	*N*,*N*-dimethylformamide; CAS RN: [68-12-2]
**DPPA**	**-**	diphenylphosphorylazide, CAS RN: [26386-88-9]
**DTBAD**	**244**	di-*tert*-butyl-azodicarboxylate; CAS-RN: [870-50-08]
**δ-OR**	**-**	δ-opioid receptor
**DPN**	-	diprenorphine, Revivon, M5050, CAS RN: [14357-78-9]
**ECAM**	**155a**	*N*^{17}-cyclopropylmethyl-azidoethylmorphine; (5*α*,6β)-6-azido-17-(cyclopropylmethyl)-4,5-epoxy-3-ethoxymorphinan; CAS RN: [93489-52-2]
**ED_{50}**	-	effective dose
**$[^{18}F]$FcyF**	**-**	$[^{18}F]$cyclofoxy, 6-deoxy-6*β*-$[^{18}F]$fluoro-naltrexone, CAS RN: [103223-58-1]
**2F-NPA**		2-fluoro-*N*-*n*-propylnorapomorphine; CAS-RN: [130434-40-1] as HBr salt
**Foxy**	**204a**	6-deoxy-6β-fluoro-oxymorphone; «fluorooxymorphone»; 4,5*α*-epoxy-6β-fluoro-17-methyl-morphinan-3,14-diol; CAS-RN: [92593-44-7]
**$[^{18}F]$foxy**	**-**	6-deoxy-6β-$[^{18}F]$fluoro-oxymorphone
**GABA**	**-**	γ-aminobutyric acid; CAS-RN: [56-12-2]
**GPCR**	**-**	G-protein coupled receptor system
**GPI**	**-**	guinea-pig ileum

Compound, Term,	Comp.	Name, Synonyms

Acronym		
**HDA reaction**	**-**	hetero Diels-Alder reaction
**4HPPDC**	**-**	4-hydroxy-phenylpyruvate decarboxylase
**IgE**	**-**	immunoglobulin E
**κ-OR**	**-**	κ-opioid receptor
**K_i**	**-**	inhibition constant
**M3G**	**-**	morphine-3-glucuronide
**M6G**	**-**	morphine-3-glucuronide
**μ-OR**	**-**	μ-opioid receptor
**M3S**	**-**	morphine-3-sulfate
**M6S**	**-**	morphine-6-sulfate
**Ms**	**-**	mesyl group
**MVD**	**-**	mouse vas deferens
**Naloxone**	**-**	*N*[17]-allyl-14-hydroxy-dihydromorphinone, *N*[17]-Allyl-noroxymorphone, CAS RN: [465-65-6]
**Naltrexone**	**-**	*N*[17]-cyclopropylmethyl-14-hydroxy-dihydromorphinone, *N*[17]-cyclopropylmethyl-noroxymorphone, NTX, CAS RN: [16590-41-3]
**NAM**	**149b**	*N*[17]-allyl-norazidomorphine; AAM; (5α,6β)-6-azido-4,5-epoxy-17-(2-propenyl)-morphinan-3-ol; CAS-RN: [56974-37-9]
**NBS**	**-**	*N*-bromosuccinimide; CAS-RN: [128-08-5]
**NCS**	**-**	*N*-chlorosuccinimide; CAS-RN: [128-09-6]
**NE**	**-**	Norepinephrine;: CAS-RN: [51-41-2]
**NEM**	**-**	*N*-ethylmaleimide; CAS RN: [128-53-0]
**NOAM**	**-**	*N*[17]-allyl-14-hydroxynorazidomorphine; (5α,6β)-6-azido-4,5-epoxy-17-(2-propenyl)-morphinan-3,14-diol; CAS-RN: [58752-76-4]
**NOP**	**-**	nociceptin/orphanin receptor
**ORs**	**-**	opioid receptors
**PBZ**	**-**	phenoxybenzamine; CAS-RN: [59-96-1]
**PET**	**-**	positron emission tomography
**PMB**	**-**	*p*-methoxy-benzyl group
**PTAD**	**-**	4-phenyl-4*H*-1,2,4-triazoline-3,5-dione; CAS-RN: [4233-33-4]
**RVD**	**-**	rat vas deferens
**SAM**	**-**	*S*-adenosylmethionine
**TBDMS**	**-**	*tert*-butyldimethylsilyl group
**TBAF**	**-**	tetrabutylammonium-fluoride; CAS-RN: [429-41-4]
**TBP**	**245**	tributylphosphine; CAS-RN: [998-40-32]
**TFA**	**-**	trifluoroacetic acid; CAS-RN: [76-05-1]
**TPP**	**-**	triphenylphosphine; CAS-RN: [603-35-0]
**TRK-130**	**278d**	naltalimide, *N*-[(5*R*,6*R*,14*S*)-17-cyclopropylmethyl-4,5-epoxy-3,14-dihydroxymorphinan-6-yl]phthalimide); CAS-RN: [160359-68-2]
**TRK-820**	**300**	nalfurafine, 17-cyclopropylmethyl-3,14-dihydroxy-4,5-epoxy-6β-[*N*-methyl-trans-3-(3-furyl)-acrylamido]morphinan, Remith®; CAS-RN: [152657-84-6]
**TyrAT**	**-**	*L*-tyrosine transaminase
**TyrDC**	**-**	*L*-tyrosine decarboxylase
**thevinone**	**172a**	4,5α-epoxy-17-methyl-3,6-dimethoxy-7α-acetyl-6,14-ethenomorphinan, CAS RN: [15358-22-2]
**TMS**	**-**	trimethylsily group
**Tos**	**-**	tosyl group
**TT**	**-**	tetanus oxide
**UGT**	**-**	5′-diphosphoglucuronyltransferase

Figure S1. Morphine alkaloid research group of the Kossuth Lajos Univesity (KLTE) in Debrecen and Alkaloida Chemical Company, Tiszavasvári, Hungary

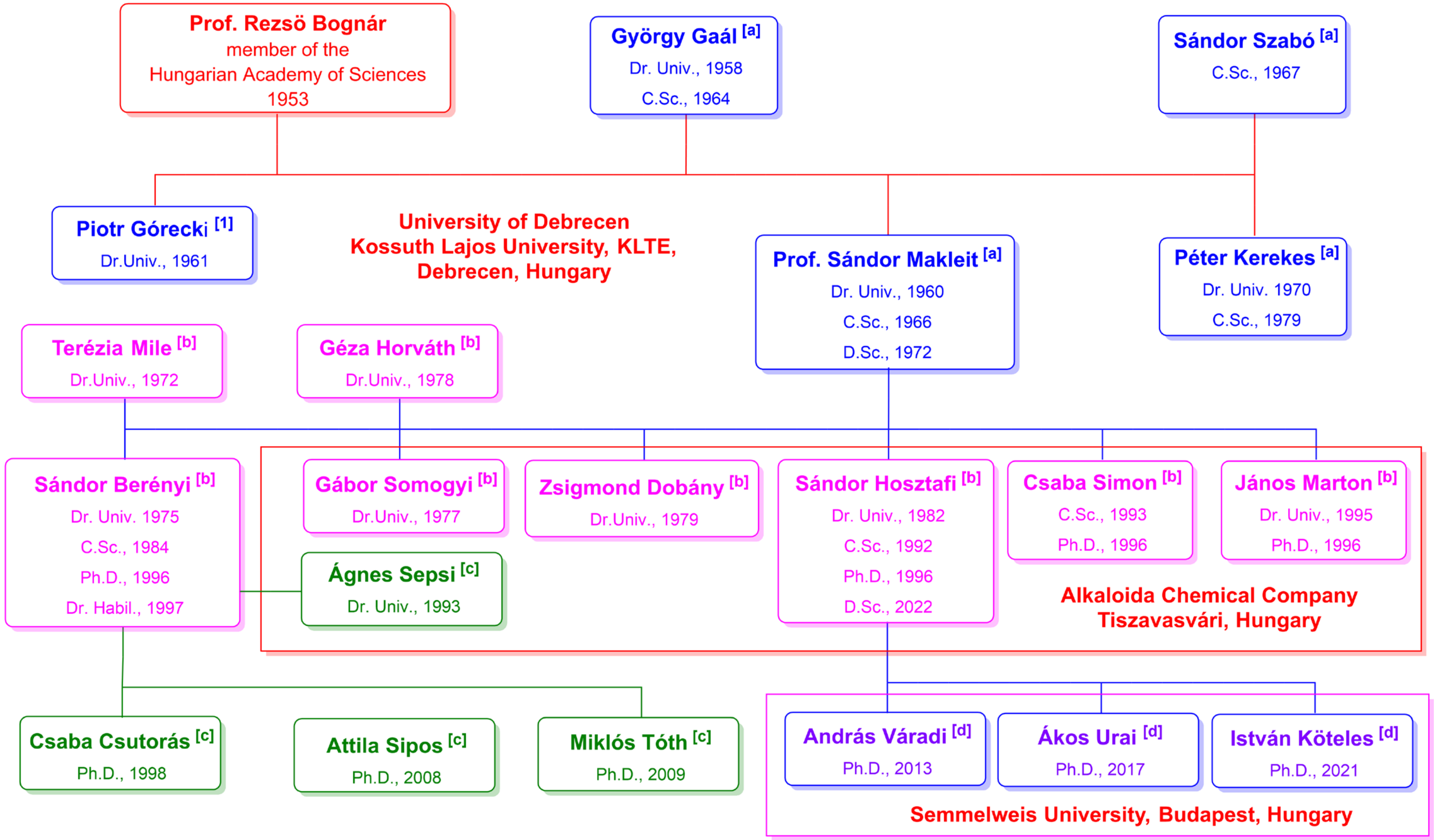

Figure 1. Scientific titles given by the KLTE in the field of poppy alkaloid research. *Supervisor*: [1] Prof. Dr. Jerzy Tułecki, Poznań, Poland; [a] Prof. Dr. Rezső Bognár, KLTE, Debrecen, Hungary; [b] Prof. Dr. Sándor Makleit, KLTE, Debrecen, Hungary; [c] Dr. Habil. Sándor Berényi, KLTE, Debrecen, Hungary; [d] Dr. Sándor Hosztafi D.Sc., Semmelweis University, Budapest, Hungary

2. Scientific titles awarded by the University of Debrecen* in the filed of poppy alkaloids [1]

* Former Kossuth Lajos University, (Kossuth Lajos Tudományegyetem, KLTE), Deberecen, Hungary

Piotr Górecki

Title: ***Dr. Univ.*** (1961)
Dissertation: *Alkaloidy uboczne słomy makowej*
Side alkaloids of poppy straw
Supervisor: Prof. Dr. Jerzy Tułecki
KLTE, Department of Organic Chemistry, Debrecen and Katedra Technologii Chemicznej Šrodków Leczniczch Akademii Medycznej, Poznań, Poland

György Gaál

Title: ***Dr. Univ.*** (1958)
Dissertation: *Kísérletek a morfinalkaloidok területén*
Experiments in the field of morphine alkaloids
Supervisor: Prof. Dr. Rezső Bognár
KLTE, Department of Organic Chemistry, Debrecen
Title: ***C.Sc.*** (1964)
Dissertation: *Mákalkaloidok kinyerésével és kémiai átalakításával kapcsolatos vizsgálatok*
Investigations regarding isolation and transformation of poppy alkaloids
Supervisor: Prof. Dr. Rezső Bognár
KLTE, Department of Organic Chemistry, Debrecen

Sándor Makleit

Title: ***Dr. Univ.*** (1960)
Dissertation: *Vizsgálatok a Solanum-alkaloidok területén*
Investigations in the field of Solanum-alkaloids
Supervisor: Prof. Dr. Rezső Bognár
KLTE, Department of Organic Chemistry, Debrecen
Title: ***C. Sc.*** (1966)
Dissertation: *Szteroidalkaloid-glikozidokkkal és szteroid-alkaloid-aglikonokkal kapcsolatos vizsgálatok*
Investigations regarding steroid-alkaloid glycosides and steroid-alkaloid aglykons
Supervisor: Prof. Dr. Rezső Bognár
KLTE, Department of Organic Chemistry, Debrecen
Title: ***D.Sc.*** (1972)
Dissertation: *Az un. morfin-sor (morfin, kodein, dihidromorfin, dihidrokodein, 14-hidroxi-kodein és 14-hidroxi-dihidrokodein) 6-O-tozil, ill. 6-O-mezil-származékai elöállítása és nukleofil szubsztitúciós reakcióinak tanulmányozása*
Preparation of the 6-*O*-tosyl- and 6-*O*-mesyl derivatives of the morphine series (morphine, codeine, dihydromorphine, 14-hydroxycodeine and 14-hydroxydihydrocodeine) and investigation of their nucleophilic substitution reactions
Doctoral Dissertation of the Hungarian Academy of Sciences (D.Sc.),
Supervisor: Prof. Dr. Rezső Bognár
KLTE, Department of Organic Chemistry, Debrecen

Szabó Sándor

Title: ***C.Sc.*** (1967)
Dissertation: *Újabb adatok a tebain redukciós termékeinek kémiájához*
Novel details to the chemistry of the reduction of thebaine
Supervisor: Prof. Dr. Rezső Bognár
KLTE, Department of Organic Chemistry, Debrecen

Péter Kerekes

Title: *Dr. Univ.* (1970)
Dissertation: *Vizsgálatok a mák ftalidizokinolin-vázas alkaloidjainak körében*
Investigations in the field of poppy alkaloids with phthalideisoquinoline skeleton
Supervisor: Prof. Dr. Rezső Bognár
KLTE, Department of Organic Chemistry, Debrecen
Title: *C.Sc.* (1979)
Dissertation: *Ftalidizokinolinvázas alkaloidok kémiai átalakítása*
Chemical transformation of phthalideisoquinoline alkaloids
Supervisor: Prof. Dr. Rezső Bognár
KLTE, Department of Organic Chemistry, Debrecen

TERÉZIA MILE

Title: *Dr. Univ.* (1972)
Dissertation: *Az allilátrendezödés mechanizmusának vizsgálata a morfin alkaloidok esetében, új származékok előállítása*
Studies on the mechanism of allylic rearrangement in case of morphine alkaloids, preparation of novel derivatives
Supervisor: Prof. Dr. Sándor Makleit
KLTE, Department of Organic Chemistry, Debrecen

SÁNDOR BERÉNYI

Title: *Dr. Univ.* (1975)
Dissertation: *Morfin alkaloidok újabb gyógyászatilag hatásos azidoszármazékainak előállítására irányuló vizsgálatok*
Investigations for the synthesis of novel, medicinally effective azido-derivatives of morphine alkaloids
Supervisor: Prof. Dr. Sándor Makleit
KLTE, Department of Organic Chemistry, Debrecen
Title: *C.Sc.* (1984), *Ph.D.* (1996)
Dissertation: *A 8,14-es helyzetben kettős kötést és kettősen allil-rendszert tartalmazó morfinszármazékok SN típusú reakciói*
SN-Type reactions of morphine derivatives containing a double bond in position 8,14 and with a double allylic system
Supervisor: Prof. Dr. Sándor Makleit
KLTE, Department of Organic Chemistry, Debrecen
Title: *Dr. habil.* (1997)
Dissertation: Morfinándiének szintézise és átalkítása opiát-, valamint dopaminreceptorokra ható vegyületekké
Synthesis of morphinandienes and their transformation to opioidergic and dopaminergic compounds

GÉZA HORVÁTH

Title: *Dr. Univ.* (1978)
Dissertation: 6-Oxomorfin alkaloidok kémiája
Chemistry of 6-oxomorphine alkaloids
Supervisor: Prof. Dr. Sándor Makleit
KLTE, Department of Organic Chemistry, Debrecen

GÁBOR SOMOGYI

Title: *Dr. Univ.* (1977)
Dissertation: *Vizsgálatok a morfin alkaloidok területén*
Investigations in the field of morphine alkaloids
Supervisor: Prof. Dr. Sándor Makleit
KLTE, Department of Organic Chemistry, Debrecen

ZSIGMOND DOBÁNY

Title: *Dr. Univ.* (1979)
Dissertation: *Neopin izolálása és hasznosítása a Kabay-féle eljárás során nyert*

	mellékalkaloid benzolból
	Utilization of neopine isolated from the accompanying-alkaloid-benzene fraction by the Kabay-process
**Supervisor**:	Prof. Dr. Sándor Makleit
	KLTE, Department of Organic Chemistry, Debrecen

Sándor Hosztafi

**Title**:	***Dr. Univ.*** (1982)
**Dissertation**:	*Morfinán és aporfin vázas vegyületek N-demetilezése*
	N-Demethylation of compounds with morphinan and aporphine skeleton
**Supervisor**:	Prof. Dr. Sándor Makleit
	KLTE, Department of Organic Chemistry, Debrecen
**Title**:	***C.Sc.*** (1992), ***Ph.D.*** (1996)
**Dissertation**:	*N-Demetilezési vizsgálatok a morfinvázas vegyületek körében*
	Investigations of the *N*-demethylation in the field of compounds with morphine skeleton
**Supervisor**:	Prof. Dr. Sándor Makleit
	KLTE, Department of Organic Chemistry, Debrecen
**Title**:	***D.Sc.*** (2022)
**Dissertation**:	*Morfinánváz C gyürüjében módosított vegyületek szintézise*
	Synthesis of in C-ring modified morphine derivatives
	Doctoral Dissertation of the Hungarian Academy of Sciences (D.Sc.), Semmelweiss University, Budapest

Csaba Simon

**Title**:	***C.Sc.*** (1993), ***Ph.D.*** (1996)
**Dissertation**:	*Mitsunobu-reakció alkalmazása a morfin-alkaloidok körében*
	Application of Mitsunobu-reaction in the field of morphine alkaloids
**Supervisor**:	Prof. Dr. Sándor Makleit
	KLTE, Department of Organic Chemistry, Debrecen

János Marton

**Title**:	***Dr. Univ.*** (1995), ***Ph.D.*** (1996)
**Dissertation**:	*Morfinándiének Diels-Alder reakciói*
	Diels-Alder reactions of morphinandienes
**Supervisor**:	Prof. Dr. Sándor Makleit
	KLTE, Department of Organic Chemistry, Debrecen

Ágnes Sepsi

**Title**:	***Dr. Univ.*** (1993)
**Dissertation**:	*Morfinándiének szintézise és Diels-Alder reakcióinak vizsgálata*
	Investigations of the synthesis of morphinadienes and their Diels-Alder reactions
**Supervisor**:	Dr. Sándor Berényi
	KLTE, Department of Organic Chemistry, Debrecen

Csaba Csutorás

**Title**:	***Ph.D.*** (1998), ***Dr. habil.*** (2009)
**Dissertation**:	*Morfinándiének szintézise és metánszulfonsavas átrendeződése*
	Synthesis of morphinandienes and their rearrangements with methanesulfonic acid
**Supervisor**:	Dr. Sándor Berényi
	KLTE, Department of Organic Chemistry, Debrecen

Attila Sipos

**Title**:	***Ph.D.*** (2008)
**Dissertation**:	*Biológiailag aktív morfinánok és aporfinok szintézise Suzuki keresztkapcsolással*
	Synthesis of pharmacologically active morphinans and aporphines with Suzuki

	reaction
**Supervisor**:	Dr. Sándor Berényi<br>KLTE, Department of Organic Chemistry, Debrecen

MIKLÓS TÓTH

**Title**:	***Ph.D.*** (2010)
**Dissertation**:	*Új kéntartalmú apomorfinok előállítása és farmakológiai vizsgálata*<br>Synthesis and pharmacological evaluation of new sulphur containing apomorphines
**Supervisor**:	Dr. Sándor Berényi<br>KLTE, Department of Organic Chemistry, Debrecen

ANDRÁS VÁRADI

**Title**:	***Ph.D.*** (2013)
**Dissertation**:	*Morfinszármazékok konjugált metabolitjainak szintézise*<br>Synthesis of conjugated metabolites of morphine
**Supervisor**:	Dr. Sándor Hosztafi, Dr. András Gergely<br>Institute of Pharmaceutical Chemistry, Semmelweis University, Budapest

ÁKOS URAI

**Title**:	***Ph.D.*** (2017)
**Dissertation**:	*6β-Acilaminomorfinánok illetve nitrogénen szubsztituált amino-alkil norvegyületek szintézise*<br>Synthesis of 6β-acylaminomorphinans and *N*-substituted *nor*-compounds
**Supervisor**:	Dr. Sándor Hosztafi<br>Institute of Pharmaceutical Chemistry, Semmelweis University, Budapest

ISTVÁN KÖTELES

**Title**:	***Ph.D.*** (2021)
**Dissertation**:	*Morfinvázas hapténej szintézise és szerkezetvizsgálata*<br>Synthesis and structural analysis of haptens with morphine skeleton
**Supervisor**:	Dr. Sándor Hosztafi<br>Institute of Pharmaceutical Chemistry, Semmelweis University, Budapest

Reference

[361] Berényi, S. A debreceni mákalkaloidkutatás 50 éve – 50 years on the Research of Poppy Alkaloids in Debrecen *MKL–Magy. Kém. Lapja*. **1999**, *54*, 548–549.
